# Evaluation of physical, chemical, and mechanical properties of a self-curing bulk-fill resin composite: An in vitro comparative study

**DOI:** 10.4317/jced.63434

**Published:** 2025-11-30

**Authors:** Maríllia Gabriella Ferreira de Souza, Emanuel Ewerton Mendonça Vasconcelos, Chiu Tzzy Haur, Natália Gomes de Oliveira, Gabriela Queiroz de Melo Monteiro, Renata Pedrosa Guimarães, Luís Felipe Espíndola-Castro

**Affiliations:** 1 DDS. School of Dentistry, Federal University of Pernambuco (UFPE), Avenida Prof. Moraes Rego, 1235 - Cidade Universitária, Recife, PE, Zip Code: 50670-901, Brazil; 2 Msc Student. School of Dentistry, Federal University of Pernambuco (UFPE), Avenida Prof. Moraes Rego, 1235 - Cidade Universitária, Recife, PE, Zip Code: 50670-901, Brazil; 3 DDS, MsC, PhD. School of Dentistry, University of Pernambuco (FOP/UPE), Avenida Norte Miguel Arraes de Alencar, 80 – Santo Amaro, Recife, PE, Zip Code: 52071-035, Brazil; 4 DDS, MSc, PhD. School of Dentistry, Federal University of Pernambuco (UFPE), Avenida Prof. Moraes Rego, 1235 - Cidade Universitária, Recife, PE, Zip Code: 50670-901, Brazil

## Abstract

**Background:**

To evaluate the physical, chemical, and mechanical properties of the Stela Automix Bulk-fill resin composite / SDI (SBF) compared to the light-cured resin composites: Filtek Bulk Fill Flow / Solventum (FBF) and Opus Bulk Fill Flow / FGM (OBF).

**Material and Methods:**

The properties assessed were microhardness, sorption, solubility, color stability, and degree of conversion. Ten samples measuring 15 mm (diameter) and 1 mm (thickness) of each material were fabricated (ISO 4049:2019). The samples were prepared and analyzed using standardized methods, including Vickers microhardness testing, sequential weighings on an analytical balance for sorption and solubility tests, CIELAB spectrophotometry for color stability assessment, and FTIR spectroscopy for determining the degree of conversion.

**Results:**

The self-curing resin composite exhibited higher microhardness (p &lt; 0.001) and degree of conversion (p = 0.009), as well as sorption and solubility values within acceptable limits, in accordance with ISO 4049:2019. However, the material exhibited greater staining in coffee, which may compromise its aesthetics.

**Conclusions:**

The self-curing resin composite tested demonstrates good overall performance and is a promising material for restorations requiring greater depth. Clinical significance: To minimize the inconvenience of pigmentation that this material undergoes, it is recommended to use it as a base material and cover it with another resin composite that offers superior color stability.

## Introduction

Resin composites are the material of choice for restoring posterior teeth (1). In this context, for restorations of deep cavities with conventional resin composite, the incremental placement technique is recommended, which involves applying the material in increments no thicker than 2 mm (2). However, this technique is not only more time-consuming but also may introduce risks of contamination and the formation of voids between resin increments (3). To minimize such drawbacks, bulk-fill resin composites were developed to simplify operative protocols, reduce the likelihood of failures, and optimize clinical time (3 , 4). The primary advantage of this material is that bulk-fill resin technology enables the fabrication of restorations in single increments of up to 5 mm, with lower polymerization shrinkage and a greater depth of cure compared to conventional resins (3 , 4). The success of this technology can be attributed to the inclusion of more effective photoinitiators in its formulation, as well as the higher translucency of bulk-fill composites, which allows improved and deeper light transmission during photopolymerization (5 , 6). However, as these systems rely on light for the polymerization process, light-induced shrinkage is to some extent unavoidable, and voids may still form between increments, potentially leading to low restoration strength (4 , 7). Furthermore, it is estimated that polymerization may be deficient in the deeper layers of the material, thereby affecting the mechanical properties of the restoration (4). Consequently, a new self-curing (chemically activated) bulk-fill composite has recently been introduced to the market, promising to improve these properties by mitigating shrinkage stress at the adhesive interfaces and enabling a greater depth of cure, even in cavities deeper than 5 mm (8). This improvement is attributed to a slower polymerization reaction and a more extended pre-gel phase, which reduces the likelihood of gap formation in the restoration (7 , 8). Thus, self-curing resins contain 70-80% by weight of borosilicate, quartz, and ceramic particles, which initiate the curing process upon mixing (8). Other components may also be part of the composition of these materials, such as irregularly shaped and macro-sized (100 m in diameter) ytterbium trifluoride and strontium fluoroaluminosilicate glass (SFASg) agglomerates, which are found in materials based on glass-ionomer cement (GIC) and calcium aluminate (7). Additionally, it has been suggested that calcium aluminate-based materials possess specific bioactive properties with the potential to remineralize the tooth-material interface and create a natural and durable seal (7). Another key feature of self-curing materials is their high mechanical strength, resulting from the greater cross-linking potential of GDMA compared to conventional materials (8). Furthermore, in the intraoral environment, restorations are subjected to cyclic loading under humid conditions with fluctuating temperature and pH, which can lead to chemical degradation of the resin composite, propagation of pre-existing cracks, and deterioration of the bonding interface (6). Similarly, physical and hydrolytic degradation can occur due to the presence of water, saliva, and acidic metabolites from cariogenic biofilm (9). These circumstances, combined, can lead to tooth fracture and marginal failure (6). Additionally, the material's surface can undergo biodegradation, which may be observed as softening and increased surface roughness (9). Furthermore, this new material is supplied with a primer free of tertiary amines, designed to act as a catalyst that initiates the polymerization process. The primer accelerates the setting reaction and enhances the degree of polymer conversion (8). Therefore, despite the improvements this new material promises, studies evaluating its physical, chemical, and mechanical properties in comparison with other materials known to have satisfactory clinical performance are still scarce. Accordingly, the objective of the present study was to evaluate the microhardness, sorption, solubility, color stability, and degree of conversion of the resin Stela Bulk Fill (SDI, Victoria, Australia) in comparison with Filtek Bulk Fill Flow (Solventum, Minnesota, USA) and Opus Bulk Fill Flow (FGM, Santa Catarina, Brazil). The null hypotheses tested were: (I) there is no difference in microhardness among the materials; (II) there is no difference in sorption and solubility among the studied materials; (III) there is no difference in the color stability of the samples; and (IV) there is no difference in the degree of conversion of the studied materials.

## Material and Methods

The self-curing bulk-fill flowable composite and the light-curing bulk-fill flowable resins evaluated in the present study are described in Table 1.

Table 1

**Figure 1:**
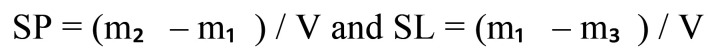
Formula.

1. Sample Preparation For the evaluation of microhardness, color stability, sorption, and solubility, ten samples of each material (n = 10) were fabricated using a split metallic mold (15 mm in diameter x 1 mm in thickness). The samples were prepared in accordance with the standards recommended by ISO 4049:2019. Photopolymerization was performed on the center of all samples using a light-emitting diode (LED) source (Quazar, FGM, Santa Catarina, Brazil) for 40 seconds on each side of the samples, with an irradiance of 1200 mW/cm². After removing the specimen from the metallic mold, excess material was removed using sequential silicon carbide abrasive papers of decreasing grit sizes (#600, 1000, and 1500). A digital caliper (± 0.01 mm; MDC-25 M, Mitutoyo, Tokyo, Japan) was used to confirm the final thickness and diameter of the specimen. Subsequently, the samples were cleaned in an ultrasonic bath (Cristófoli, Paraná, Brazil) with distilled water for 10 minutes and gently dried with filter paper. A single operator prepared the samples, and a blinded evaluator performed the analyses described below: 2. Vickers Microhardness Analysis The Vickers microhardness (VHN) test was conducted using a digital microhardness tester (ISH-MR 150/ INSIZE, São Paulo, Brazil). The indenter was positioned centrally on the specimen (n = 10), and a load of 300 gf was applied for 15s. Subsequently, three indentations were made per specimen, with a 5 mm distance between them. The average of the three measurements was calculated and used as the microhardness number for the specimen (1). 3. Sorption and Solubility Assessment The samples (n = 5, recommended by the ISO 4049:2019 standard) were placed in a desiccator containing silica gel at 37 ± 2°C. After 24 hours, the samples were kept at 23 ± 2°C for an additional 2 hours. Subsequently, all samples were weighed on an analytical balance (±0.01 mg; AUW 220D, Shimadzu Analytical Balance, Tokyo, Japan) repeatedly until a constant mass was obtained (m1), with a variation of less than 0.1 mg. The diameter and thickness of each specimen were measured at four points using a digital caliper (± 0.01 mm; MDC-25 M, Mitutoyo, Tokyo, Japan). The samples were stored in 50 ml Falcon tubes containing distilled water and kept at 37°C for 7 days. Afterward, they were removed, gently dried with filter paper, weighed again, and this mass was recorded (m2). The samples were returned to the desiccator and weighed repeatedly (daily) until a constant mass was reached (m3). Sorption (SP) and solubility (SL) (in µg/mm³) were calculated using the following equations, (Fig. 1):

1

**Figure 2:**
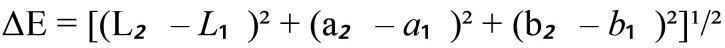
Formula.

Where: The volume of the samples was calculated in mm³ (V=r²h), where r is the mean radius of the specimen (diameter/2) and h is the mean thickness of the specimen. 4. Color Stability Color analysis was performed by a single operator at three different time points: (1) before immersion, (2) after 1 day, and (3) after 1 week of storage in either distilled water or coffee. The five samples (n = 5) immersed in distilled water for the sorption and solubility tests served as the control group. The other five samples, which had undergone the Vickers microhardness test, were immersed in coffee. The coffee solution was prepared by dissolving 0.51 g of instant coffee powder in 50 mL of distilled water (Nescafé, Nestlé, São Paulo, SP, Brazil). The CIELAB system is composed of three axes: L* (lightness, from 0 = black to 100 = white), a* (from -a = green to +a = red), and b* (from -b = blue to +b = yellow). The color change (E) was calculated according to the equation, (Fig. 2):

2

**Figure 3:**
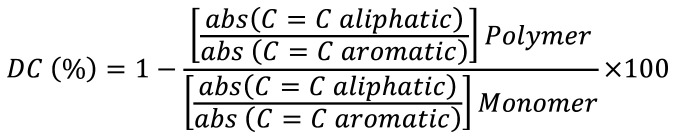
Formula.

Where: The subscripts 1 and 2 refer to the initial and final color readings, respectively. Two readings were taken from the central area of each specimen, and the average values were used for analysis (1). 5. Degree of Conversion Analysis Five samples per group were fabricated (5 mm in diameter and 2 mm in thickness). Before and after polymerization, the resins were analyzed using FTIR spectroscopy. Each specimen was scanned 32 times over a wavelength range of 4000 to 400 cm-¹ with a resolution of 4 cm-¹. All readings were taken from the center of the top surface of the specimen (in contact with the polyester strip) in contact with the diamond of the ATR unit. The degree of conversion (DC) was analyzed based on the ratio of the aliphatic carbon-carbon double bond to the aromatic group for unpolymerized and polymerized samples, respectively, according to the following equation, (Fig. 3):

3

Where the degree of conversion (DC) is determined by the absorbance peak of the aliphatic C=C bonds at a wavelength of 1637 cm-¹ and the absorbance peak of the aromatic bonds at 1607 cm-¹ for the tested resin composites. The equation is based on the ratio between the polymerized (polymer) and unpolymerized (monomer) components of the resin composite (4). The polymerized resins were analyzed at two time points: two hours after polymerization and after seven days, when stored dry in dark plastic containers in an incubator at 37°C (± 1°C). 6. Statistical Analysis The sample data were analyzed descriptively using means and standard deviations. For the comparison among the resins, the F-test (ANOVA) was applied, taking into account the normality of the data. The Mann-Whitney test was used for the comparison between solutions. The paired Wilcoxon test was performed to compare the two evaluation times for each material group. When the F-test (ANOVA) indicated a significant difference, multiple comparisons were performed using the Tukey test. For differences in the Kruskal-Wallis test, comparisons were made using the Conover test. The choice of tests considered the normality (Shapiro-Wilk) and homogeneity of variances (Levene's test). A significance level of 5% was adopted, and the analyses were performed using IBM SPSS version 27 and MedCalc version 20.104 software.

## Results

In the microhardness analysis, higher values were observed for the SBF resin (mean 36.40), followed by OBF (mean 32.88) and FBF (mean 32.00), as shown in Table 2.

Table 2

These results show a significant difference (p &lt; 0.001) among the studied materials. Table 3 presents the mean values for the sorption analysis among the resin samples. SBF (mean 24.23 µg/mm³) exhibited the highest sorption value, compared to OBF (mean 18.68 µg/mm³) and FBF (mean 15.29 µg/mm³).

Table 3

For solubility, Table 3 indicates that there was no significant difference among the values. However, the OBF resin (mean 1.92 µg/mm³) presented the highest solubility, while the mean values for SBF and FBF were 1.36 µg/mm³ and 1.02 µg/mm³, respectively. Statistically significant differences (p&lt;0.05) were found among the groups regarding sorption; for solubility, the most important statistical difference was observed between OBF and FBF. Tables 4 and 5 present the statistical data for color stability on the first day (E1) and the seventh day (E7).

Table 4

Table 5

In Table 4, the E1 was greater for the coffee solution than for distilled water for all analyzed materials. Comparatively, SBF showed the highest mean E1 in coffee (16.24) and the lowest in water (0.64). On the other hand, OBF showed a higher mean value in distilled water (1.61) and a mean of 10.21 in the coffee solution. As for FBF, it exhibited the most excellent color stability (lowest E1) in coffee (7.13) after one day, with a mean of 0.86 in distilled water. Table 5 shows the color stability values at seven days in the coffee solution and in distilled water. The pattern of greater staining in coffee than in water was maintained. The SBF material showed the highest mean value (i.e., most staining) in coffee (20.09), followed by OBF (13.78), and finally FBF (9.29), which had the best color stability. In distilled water, SBF (0.49) best preserved its color, followed by FBF (1.06) and OBF (1.40). The degree of conversion, with values at two hours and seven days, was analyzed in Table 6.

Table 6

All groups showed a higher degree of conversion after one week, with the highest results observed for the SBF resin, both at two hours (77.63) and at one week (90.87). This was followed by OBF, with values of 66.17 at two hours and 73.90 at one week. The lowest values were observed in the FBF group, with 65.15 at two hours and 72.56 at seven days. The statistical analysis showed a significant difference (p &lt; 0.05) among the three groups, particularly between SBF and the other resins.

## Discussion

The first null hypothesis, which stated there would be no differences in the microhardness of the materials, was rejected. According to Table 2, the SBF resin exhibited higher microhardness compared to FBF and OBF. This characteristic is related to the high degree of conversion observed in chemically activated restorative materials, which demonstrate higher monomer polymerization rates than light-cured resin composites, whose conversion can range from 35% to 77% (10). Furthermore, one study highlighted ytterbium trifluoride (YbF3), present in the SBF group, as a potential functional additive that enhances mechanical properties and contributes to an increase in the resin's cross-linking density (11). The study of microhardness is crucial for estimating the durability of restorative material in teeth. Microhardness analyses suggest the level of wear, polish, and abrasion that a material can exert on teeth (12). In line with this idea, a resin composite with good mechanical properties is likely to withstand masticatory forces (10). However, photopolymerization does not completely convert the monomers present in the composite, which can limit the material's degree of microhardness (10). The second null hypothesis, regarding sorption and solubility, was partially supported. For sorption, there were relevant differences in the results; however, for solubility, the present study did not identify differences in the values obtained from the samples. That said, the SBF composite exhibited the highest sorption compared to the other analyzed materials, as shown in Table 3. The ISO 4049:2019 standard specifies a sorption limit of 40 µg/mm³ for storage in water; therefore, all materials studied are within the recommended standards (13). Evaluating the sorption and solubility of dental restorative materials is of utmost importance for understanding the properties of the material, as well as being aware of its longevity and failure rates (14). A study has shown that sorption can be influenced by the resin's degree of porosity, the nature of the filler, and the filler-matrix interface of the material. Nevertheless, these factors can directly affect the amount of solvent absorbed, particularly with extended exposure times. In the same study, the author states that sorption is carried out by diffusion and is regulated by two mechanisms of action: the incorporation of solvent molecules at the filler/matrix interface and also in the intermolecular spaces; and the interrelation between solvent molecules and hydrophilic groups to form hydrogen bonds (14). The solubility rate of restorative materials is directly related to the polymerization time adopted (15). Recent studies suggest that inefficient photopolymerization can lead to increased sorption and solubility of the material, thereby promoting biomechanical degradation, resin staining, microleakage, and the development of secondary caries (15). Light-cured resin composites are particularly more susceptible to the elution of residual monomers, especially when subjected to accelerated polymerization protocols, such as those of 3s, 5s, 10s, or 20s (15). Additionally, composites with greater polymer chain rigidity may exhibit high concentrations of unpolymerized monomers, which favors their leaching and consequently increases the solubility level (16). In a recent study, the most frequently eluted monomer was bisphenol A glycidyl methacrylate (Bis-GMA), whereas another study highlighted triethylene glycol dimethacrylate (TEGDMA) due to its low molecular weight (15 , 16). There are also indications that filler nanoparticles may also be eluted from the composite (16). According to Table 3, there was no significant difference in the solubility results of the studied materials. This result can be attributed to the methodology used in this study, which employed a photopolymerization protocol for the FBF and OBF resins that involved a longer exposure time (40 seconds on each side of the specimen). Thus, it is likely that the monomer conversion rate was sufficiently high to prevent a significant degree of elution, which would justify the similarity of the results obtained in relation to the SBF resin. Therefore, the material must exhibit stability in the oral cavity to minimize undesirable clinical effects, such as allergic reactions and marginal failures, preserving the function and health of the patient using these dental materials (17). According to the ISO 4049:2019 standard, the maximum acceptable solubility limit is 7.5 µg/mm³; thus, the resins analyzed in this study comply with the parameters established by this standard (13). The third null hypothesis was rejected, as it stated that there would be no differences in color stability among the resin composites, which is evident in Table 4. After one day of immersion in water, the OBF resin (1.61) had the highest result, followed by FBF (0.86) and SBF (0.64). In coffee, SBF (16.24) had the highest values, followed by OBF (10.21) and FBF (7.13), respectively. These results are consistent with some current studies (1 , 18 , 19). Furthermore, the interpretation of E values is fundamental for maintaining clinical and aesthetic control of the color observed in the composite resin (20). Therefore, E values &lt;1 are hardly perceptible to people with accurate vision; conversely, a E 3.3 becomes noticeable to the human eye. Thus, a clinically acceptable E varies between 1 and 3.3 (1 , 20). In this research, a digital spectrophotometer was used to measure the color after one and seven days of immersion in distilled water and coffee. Thus, dissatisfaction with the aesthetic appearance of dental restorations represents one of the main reasons for failures in anterior restorations. In this context, maintaining the color of resin composites is fundamental to the aesthetic effectiveness of these restorative interventions (18). Color change in restorations is associated with both intrinsic and extrinsic factors (1 , 19). Extrinsic factors include colorants present in foods, such as coffee, tea, alcoholic beverages, and juices, as well as nicotine from cigarettes, which undergo a sorption process and adhere to the restorative material (1). In conjunction, water from saliva is absorbed by the resin, and over time, this can promote the emergence of microcracks or interfacial spaces between the resin matrix and filler particles. This leads to greater permeability of the colorant into the resin composites (18). Additionally, there may be an exchange of water molecules already absorbed by the resin composite for pigment molecules, or this water may even act as a carrier, entering the resin and clinically causing stains (18). At one week, Table 5 shows that the SBF (0.49) and OBF (1.40) groups immersed in water presented a slight decrease in values. In contrast, FBF (1.06) showed an increase, highlighting that color stability can vary depending on the composition of the restorative material and the polymer formed. On the other hand, immersion in coffee for one week resulted in an increase in E values for all analyzed samples, with SBF (20.09) exhibiting the most staining, followed by OBF (13.78) and FBF (9.29), as shown in Table 5. The yellow pigments contained in coffee interact easily with the organic phase of the resin, which favors the binding of the colorant to the resin (18). It is worth noting that the hydrogen potential (pH) of foods or beverages can lead to the deterioration of the resin, resulting in surface irregularities and facilitating the absorption of coloring agents (1 , 20). The types of organic matrix are also related to the staining of composite resins, as monomers like UDMA, present in SBF and FBF, are more resistant to water absorption and color change compared to Bis-GMA. However, a low filler content promotes chromatic instability in the resin (21). This issue is observed in the present study, as the SBF group was the most pigmented, despite containing UDMA in its composition; however, the resin has a low filler content (21). It is essential to highlight that, regarding pigmentation, the present study tested the restorative materials under extreme conditions, with immersions in coffee for up to 7 days. In a clinical trial evaluating Stela Bulk Fill after 18 months, compared to a conventional bulk-fill resin (Filtek One / Solventum), it was observed that the self-curing composite exhibited functional and biological performance comparable to that of the light-cured bulk-fill composite (22). The fourth null hypothesis, which stated that there are no differences in the degree of conversion among the studied groups, was rejected. Table 6 shows the results of this research regarding the degree of conversion of the groups at two hours and one week, with SBF showing superior values at both time points. Based on this, in two hours, SBF (77.63) surpassed the results of OBF (66.17) and FBF (65.15). Likewise, at one week, SBF (90.87) presented very satisfactory results compared to OBF (73.90) and FBF (72.56). The degree of conversion of restorative composites influences several key characteristics, including microhardness, sorption, solubility, and cytotoxicity (17). Because SBF is self-curing, meaning it does not require light, it exhibits a high polymerization rate and, consequently, a higher degree of conversion (11). A study also evaluated the polymerization conversion of Stela Bulk Fill after 720 s, reporting a degree of conversion of 57.7% (8). This finding indicates that the material continues to polymerize over time. The same study showed that when the primer was applied together with Stela Bulk Fill, it significantly increased the degree of conversion, suggesting that clinical polymerization may be even higher. Similarly, the relationship between polymerization shrinkage and porosity is inversely proportional (11). Effective polymeric conversion promotes fewer gaps between the matrix and filler, reduces voids in the composite, and provides a denser structure (11). In contrast, it has been observed that light-cured composite resins present greater challenges in the polymeric conversion of their monomers, where Bis-GMA was the most eluted from the samples due to incomplete conversion of the double bond, resulting from phenomena such as vitrification, gelation, immobilization, and steric hindrance (10). The limitations of an in vitro study include factors such as the absence of real clinical conditions that directly influence the study material, including saliva pH, biofilm, and masticatory load. The short analysis time of the research samples also influences the results obtained, which limits the data for the material's medium and long-term performance. The fact that the Stela Primer was not used in the research can also be considered a limitation, as its clinical use could have improved the adhesion between the resin and the substrate, potentially contributing to better mechanical stability of the material. Considering the results obtained, it is recommended that further studies be conducted to address variables not covered in this research. Analyses with larger samples, a prolonged storage period, and studies associating the Stela Automix resin with its respective adhesive, as well as investigations involving human teeth, could provide a more comprehensive understanding of the behavior of the studied resin.

## Conclusions

The self-curing Bulk Fill Flow resin composite (Stela Automix / SDI) showed performance equal to or exceeding that of conventional resin composites in terms of microhardness, sorption, solubility, and degree of conversion. However, its color stability when exposed to pigmented foods still represents a limitation. Therefore, it is recommended that, whenever possible, the resin be overlaid with a conventional light-cured resin composite to preserve its long-term aesthetic properties.

## Figures and Tables

**Table 1:** The researched resin composites and their compositions.

GROUP	RESIN	COMPOSITION	BATCH
SBF	Stela Automix Bulk Fill (SDI, Victoria, Australia)	UDMA, GDMA, fumed silica, barium aluminoborosilicate glass (mean particle size of 2.8 µm; distribution range of approximately 2 to 5 µm), fluoroaluminosilicate glass (mean particle size of 4.0 µm; distribution range of roughly 2 to 8 µm), ytterbium trifluoride (YbF₃), calcium aluminate, hydroperoxide-based initiators, stabilizers, pigments. Filler particle content: 61% by weight and 36% by volume.	1220605
FBF	Filtek Bulk Fill Flow (Solventum, Minnesota, EUA)	Silane-treated ceramic, substituted dimethacrylate, Bisphenol A diglycidyl dimethacrylate, silane-treated silica, TEGDMA, ytterbium fluoride, reacted polycaprolactone polymer, diphenyliodonium hexafluorophosphate. Filler particle content: 76.5% by weight and 58.5% by volume.	NF18261
OBF	Opus Bulk Fill Flow (FGM, Santa Catarina, Brazil)	Urethane dimethacrylate monomers, photoinitiators and APS co-initiators, silicon dioxide (silica), stabilizers, pigments. Filler particle content: 79% by weight.	090822

UDMA: Urethane dimethacrylate; GDMA: Glycerol dimethacrylate; TEGDMA: Triethylene glycol dimethacrylate.

**Table 2:** Evaluation of the microhardness of resin composites based on mean and standard deviation (SD).

Material	StatisticsMean ± SD
SBF	36.40 ± 2.94 (A)
FBF	32.00 ± 2.14 (B)
OBF	32.88 ± 1.59 (B)
p-value	p (1) < 0.001*

(*) Significant difference at the 5% level of significance. (1) F-test (ANOVA) with Tukey’s post-hoc comparisons. Note: Means followed by different letters in parentheses are significantly different.

**Table 3:** Evaluation of the sorption and solubility of resin composites based on mean and standard deviation.

Material	Sorption	Solubility
	Mean ± SD	Mean ± SD
SBF	24.23 ± 1.57 (A)	1.36 ± 0.31 (AB)
FBF	15.29 ± 0.57 (B)	1.02 ± 0.47 (A)
OBF	18.68 ± 2.53 (C)	1.92 ± 0.50 (B)
p-value	p (1) = 0.003*	p (1) = 0.044*

(*) Significant difference at the 5% level of significance. (1) Kruskal-Wallis test with Conover’s post-hoc comparisons. Note: Means in the same column followed by different letters in parentheses are significantly different.

**Table 4:** Color evaluation using a spectrophotometer after 1 day of immersion in water and coffee (ΔE1).

Material	Solution		p-value
	Distilled water	Coffee	
	Mean ± SD	Mean ± SD	
SBF	0.64 ± 0.19	16.24 ± 4.08 (A)	p (1) = 0.009*
FBF	0.86 ± 0.31	7.13 ± 0.92 (B)	p (1) = 0.000*
OBF	1.61 ± 0.90	10.21 ± 1.37 (C)	p (1) = 0.009*
p-value	p (2) = 0.105	p (2) = 0.003*	

(*) Significant difference at the 5% level of significance. (1) Mann-Whitney test. (2) Kruskal-Wallis test with Conover’s multiple comparisons. Note: Means in the same column followed by different letters in parentheses are significantly different.

**Table 5:** Color evaluation using a spectrophotometer after 7 days of immersion in water and coffee (ΔE7).

Material	Solution		p-value
	Distilled water	Coffee	
	Mean ± SD	Mean ± SD	
SBF	0.49 ± 0.30	20.09 ± 4.93 (A)	p (1) = 0.009*
FBF	1.06 ± 0.67	9.29 ± 1.26 (B)	p (1) = 0.009*
OBF	1.40 ± 0.19	13.78 ± 1.52 (C)	p (1) = 0.009*
p-value	p (2) = 0.069	p (2) = 0.004*	

(*) Significant difference at the 5% level of significance. (1) Mann-Whitney test. (2) Kruskal-Wallis test with Conover’s multiple comparisons. Note: Means in the same column followed by different letters in parentheses are significantly different.

**Table 6:** Evaluation of the degree of conversion of resin composites at two hours and one week after curing.

Material	Time for evaluation		p-value
	Two Hours	One Week	
	Mean ± SD	Mean ± SD	
SBF	77.63 ± 2.03 (A)	90.87 ± 0.83 (A)	p (1) = 0.043*
FBF	65.15 ± 1.82 (B)	72.56 ± 1.59 (B)	p (1) = 0.043*
OBF	66.17 ± 1.78 (B)	73.90 ± 2.36 (B)	p (1) = 0.043*
p-value	p (2) = 0.007*	p (2) = 0.009*	

(*) Significant difference at the 5% level of significance. (1) Paired Wilcoxon test. (2) Kruskal-Wallis test with Conover’s multiple comparisons. Note: Means in the same column followed by different letters in parentheses are significantly different.

## Data Availability

Available upon request.

## References

[1] Espíndola-Castro LF, Durão MA, Pereira TV, Cordeiro AKB, Monteiro GQM (2020). Evaluation of microhardness, sorption, solubility, and color stability of bulk fill resins: A comparative study. J Clin Exp Dent.

[2] Melo RA, Bispo ASL, Barbosa GAS, Galvão MR, Assunção IV, Souza RODA (2019). Morphochemical characterization, microhardness, water sorption, and solubility of regular viscosity bulk fill and traditional composite resins. Microsc Res Tech.

[3] Besegato JF, Jussiani EI, Andrello AC, Fernandes RV, Salomão FM, Vicentin BLS (2019). Effect of light-curing protocols on the mechanical behavior of bulk-fill resin composites. J Mech Behav Biomed Mater.

[4] Sampaio CS, Abreu JLB, Kornfeld B, Giannini M, Hirata R (2024). Short curing time bulk fill composite systems: volumetric shrinkage, degree of conversion and Vickers hardness. Braz Oral Res.

[5] Gomes de Araújo-Neto V, Sebold M, Fernandes EC, Feitosa VP, Giannini M (2021). Evaluation of physico-mechanical properties and filler particles characterization of conventional, bulk-fill, and bioactive resin-based composites. J Mech Behav Biomed Mater.

[6] Lopes MWP, Borba M, Bortoluzzi A, Bervian J, Collares KF (2023). Fatigue and marginal adaptation of bulk fill restoratives: Effect of the layering technique and cavity dimension of extensively damaged teeth. Dent Mater.

[7] Pires PM, Neves AA, Lukomska-Szymanska M, Farrar P, Cascales AF, Sauro S (2024). Bonding performance and interfacial adaptation of modern bulk-fill restorative composites after aging in artificial saliva: an in vitro study. Clin Oral Investig.

[8] Guarneri JAG, Maucoski C, Ghaffari S, Macneil BD, Price RB, Arrais CAG (2025). Ability of a novel primer to enhance the polymerization of a self-cured resin composite. Dent Mater.

[9] Brito O, Oliveira I, Monteiro GQM (2019). Hydrolytic and Biological Degradation of Bulk-fill and Self-adhering Resin Composites. Oper Dent.

[10] Moldovan M, Balazsi R, Soanca A, Roman A, Sarosi C, Prodan D (2019). Evaluation of the Degree of Conversion, Residual Monomers, and Mechanical Properties of Some Light-Cured Dental Resin Composites. Materials (Basel).

[11] Laporte C, Bourgi R, Jmal H, Ben Ammar T, Hazko S, Addiego F (2025). Mechanical, Antibacterial, and Physico-Chemical Properties of Three Different Polymer-Based Direct Restorative Materials: An In Vitro Study. Polymers (Basel).

[12] Elhejazi AA, Alosimi A, Alarifi F (2024). The effect of depth of cure on microhardness between bulk-fill and hybrid composite resin material. Saudi Dent J.

[13] (2019). Report No. Dent - Polym-Based Restor Mater.

[14] Alzahrani B, Alshabib A, Awlyia W (2023). The depth of cure, sorption, and solubility of dual-cured bulk-fill restorative. Materials.

[15] Thanoon H, Silikas N, Watts DC (2024). Effect of polymerization protocols on water sorption, solubility, and hygroscopic expansion of a fast-cure bulk-fill composite. Dent Mater.

[16] Klarić N, Macan M, Par M, Tarle Z, Marović D (2022). Effect of Rapid Polymerization on Water Sorption and Solubility of Bulk-fill Composites. Acta Stomatol Croat.

[17] Öztürk ANS, Harorli OT (2024). Bulk-fill composite in challenging cavities: conversion rate, solubility, and water absorption analysis. Odontology.

[18] Ma X, Zhang X, Huang X, Liu F, He J, Mai S (2024). Performance of low shrinkage Bis-EFMA based bulk-fill dental resin composites. Dent Mater.

[19] Bai X, Chen Y, Zhou T, Pow EHN, Tsoi JKH (2024). The chemical and optical stability evaluation of injectable restorative materials under wet challenge. J Dent.

[20] Oliveira LS, Silva J, Silva L, Leal CMB, Regalado DF, Toda C (2024). Effects of different beverages on the color stability and fluorescence of resin composites: in situ study. Clin Oral Invest.

[21] Ömeroğlu MK, Hekimoğlu HC (2025). Evaluation of colour stability, water sorption, and solubility of no-cap flowable bulk fill resin composites. BMC Oral Health.

[22] Loguercio AD, Carpio-Salvatierra B, Ñaupari-Villasante R, Armas-Vega A, Cavagnaro S (2025). Clinical Evaluation of a New Chemically-Cured Bulk-Fill Composite in Posterior Restorations: 18-Month Multicenter Double-Blind Randomized Clinical Trial. J Dent.

